# Single-Cell Sequencing Applications in the Inner Ear

**DOI:** 10.3389/fcell.2021.637779

**Published:** 2021-02-12

**Authors:** Mingxuan Wu, Mingyu Xia, Wenyan Li, Huawei Li

**Affiliations:** ^1^ENT Institute and Department of Otorhinolaryngology, Eye and ENT Hospital, State Key Laboratory of Medical Neurobiology and MOE Frontiers Center for Brain Science, Fudan University, Shanghai, China; ^2^Institutes of Biomedical Sciences, Fudan University, Shanghai, China; ^3^NHC Key Laboratory of Hearing Medicine, Fudan University, Shanghai, China; ^4^The Institutes of Brain Science and The Collaborative Innovation Center for Brain Science, Fudan University, Shanghai, China

**Keywords:** single cell sequencing, hair cell, inner ear, supporting cell, spiral ganglion neuron

## Abstract

Genomics studies face specific challenges in the inner ear due to the multiple types and limited amounts of inner ear cells that are arranged in a very delicate structure. However, advances in single-cell sequencing (SCS) technology have made it possible to analyze gene expression variations across different cell types as well as within specific cell groups that were previously considered to be homogeneous. In this review, we summarize recent advances in inner ear research brought about by the use of SCS that have delineated tissue heterogeneity, identified unknown cell subtypes, discovered novel cell markers, and revealed dynamic signaling pathways during development. SCS opens up new avenues for inner ear research, and the potential of the technology is only beginning to be explored.

## Introduction

The inner ear is one of the most intricate parts of the body, located within the petrous portion of the temporal bone with its attendant sensory structures responsible for auditory and balance function (Groves and Fekete, 2012). The cochlea is the auditory portion of the membranous labyrinth, while the vestibular organ collects motion, equilibrium, and spatial orientation information. Previous studies have defined some crucial signaling pathways – such as FGF, Notch, and Wnt – and genes – such as *Pax2* and *Atoh1* – that play essential roles in the development and maintenance of the inner ear (Scheffer et al., 2015; Zhong et al., 2019). Each region of the sensory epithelium is composed of highly heterogeneous populations of cells depending on the physiological and anatomical criteria of that region (Szarama et al., 2012; Jeng et al., 2020; Ono et al., 2020; Yao et al., 2020), and this highlights the importance of examining the expression of a large number of genes at the single cell level.

Previous genetic studies have mainly focused on analyzing bulk tissue samples composed of millions of cells, and thus have only looked at the average expression of specific transcripts, which is dependent on the expression level of each gene as well as the populations of different cell subtype. Over the past centuries, major advances have been made in genomic studies, and single cell sequencing (SCS) has emerged as a powerful tool for studying the contributions from individual cells (Knouse et al., 2014; Cao et al., 2019; Pijuan-Sala et al., 2019). In this review, we will briefly show the developmental history and utilization of SCS technology in basic research, and we will summarize its utility in inner ear research that has improved our knowledge of inner ear cellular heterogeneity, inner ear development, genetic deafness, and hair cell (HC) regeneration.

## The Development and the Advantages of SCS Technology

**FIGURE 1:**
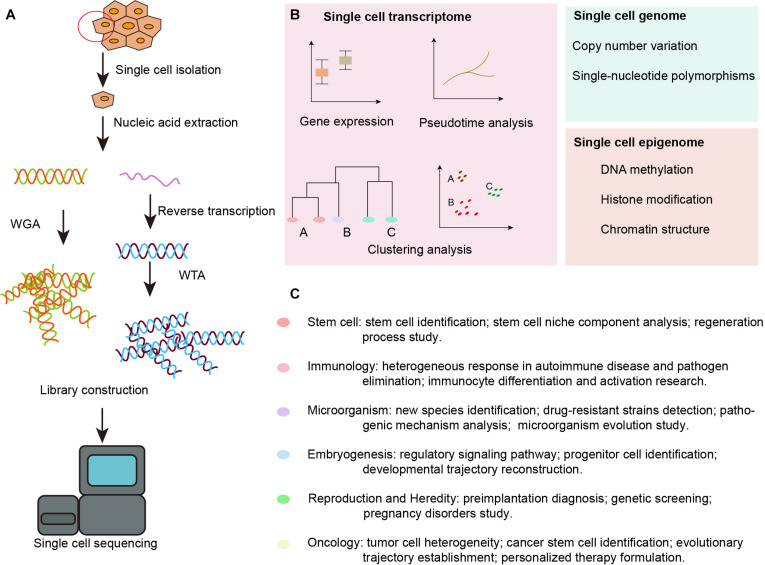
Overview of SCS workflow and applications in the inner ear. **(A)** Schematic strategy of SCS workflow. After dissociation of the organ or tissue of interest to live single cell, cells are then captured by various methods and lysed to release RNA and DNA fragment, the former is reversed transcribed to synthesized cDNA. DNA fragment or cDNA must be amplified to generate sequencing library. Next-generation sequencing is subsequently performed to generate the readouts that can be assigned to single cells via cell-specific barcodes. **(B)** Analysis of single cell transcriptome, genome and epigenome data. **(C)** Diverse fields of basic research that have been impacted by SCS technologies. WGA, whole genome amplification; WTA, whole transcriptome amplification.

With the advent of next-generation sequencing, SCS technology has been developed and adopted to obtain genomic, transcriptomic, and epigenomic information from single cells (Tang X. et al., 2019). The data are collected following single cell isolation, capture, and lysis, nucleic acid extraction and amplification, and high-throughput sequencing, as shown in Figure 1A. Tang et al. (2009) modified the previously reported single-cell transcriptome amplification method and analyzed the transcriptomes of individual blastomeres, and this marked the beginning of single-cell omics. Later, Navin et al. (2011) flow-sorted nuclei and investigated copy number variations in liver tumor subpopulations. Further, Smart-Seq was developed as a robust method for improving read coverage and for enhancing the detailed analyses of alternative transcript isoforms and the identification of single-nucleotide polymorphisms (Ramskold et al., 2012).

The SCS procedures have been continuously updated and modified over the past decade. Advances in single-cell isolation have greatly expanded the fields of research, as cells of various tissue could be isolated and captured by different method. Magnetic-activated cell sorting (MACS), flow-activated cell sorting (FACS), and microfluidic platforms enable high throughput study, while laser capture microdissection (LCM) technology preserves original spatial information which may be of great importance under some circumstances. In addition, numerous methods have been developed for single cell genome, transcriptome, and epigenome study, as shown in Figure 1B. Coverage, sensitivity, efficiency as well as accuracy of these methods differ from each other because of distinct amplification process. Characteristics and suitable applications of different methods are summarized as shown in Tables 1–4.

**TABLE 1:** Comparison of single-cell isolation methods.

Item	Advantage	Disadvantage	References
Serial dilution	Simple operation, low cost	Low throughput, cell loss, difficulty in filtering target cells	Gross et al., 2015
Manual pipetting	Simple operation, low cost	Low throughput	Gross et al., 2015
Robotic micromanipulation	Precise separation	Low efficiency, dependence on apparatus, mechanical damage	Guo et al., 2017a
MACS	High throughput, high accuracy	Mechanical damage, requirement for high cell abundance	Guo and Cairns, 2019
FACS	High throughput, high accuracy	Mechanical damage, requirement for high cell abundance and fluorescence intensity	Shapiro et al., 2013
LCM	High accuracy, conservation of spatial information	Low efficiency, high cost, likely contamination form adjacent cells	Nichterwitz et al., 2016
Microfluidic platforms	High throughput, high accuracy, available commercial platform	Dependence on homogeneous cell size	Macosko et al., 2015

*MACS, magnetic-activated cell sorting; FACS, flow-activated cell sorting; LCM, laser capture microdissection.*

**TABLE 2:** Comparison of single-cell DNA sequencing technologies.

Item	Coverage	Characteristics	Application	References
DOP-PCR	Low	Exponential amplification, simple operation, fast speed, sequence-dependent bias, high allele dropout rate	CNV	Arneson et al., 2008; Huang et al., 2015
MDA	Medium	Exponential amplification, high replication fidelity, sequence-dependent bias, normalization ineffective	SNV	Spits et al., 2006
MALBAC	High	Linear amplification, high accuracy for CNV detection, low false negative rate for SNV detection	CNV and SNV	Zong et al., 2012

*DOP-PCR, degenerate oligonucleotide–primed polymerase chain reaction; MDA, multiple displacement amplification; MALBAC, multiple annealing and looping-based amplification cycles; CNV, copy number variation; SNV, single-nucleotide polymorphisms.*

**TABLE 3:** Comparison of single-cell RNA sequencing technologies.

Item	Coverage	Characteristics	References
Smart-seq/seq2	Full length	High sensitivity, low efficiency	Ramskold et al., 2012; Picelli et al., 2014
Quarz-seq/seq2	Full length	High sensitivity, high reproducibility	Sasagawa et al., 2013, 2018
CEL- seq/seq2	Full length	High accuracy, sequence-dependent bias	Hashimshony et al., 2012, 2016
Drop-seq	3′-end	High efficiency, low cost, highly parallel analysis	Macosko et al., 2015
inDROP-seq	3′-end	High throughput, low efficiency	Klein et al., 2015
STRT-seq	5′-end	High accuracy, sequence-dependent bias	Islam et al., 2012
MARS-seq	3′-end	High sensitivity, high accuracy	Jaitin et al., 2014
Cyto-seq	3′-end	Direct analysis of complex samples, relatively expensive and time-consuming	Fan et al., 2015

**TABLE 4:** Comparison of single-cell epigenome technologies.

Item	Application	Characteristics	References
scRRBS-seq	DNA methylation	Low throughput, low coverage rate	Guo H. et al., 2015
scBS-seq	DNA methylation	Low throughput, low coverage rate	Smallwood et al., 2014
scCGI-seq	DNA methylation	Low throughput, high coverage rate	Han et al., 2017
scChIL-seq	Histone modification	Low throughput, high coverage rate	Harada et al., 2019
scCUT&tag	Histone modification	High throughput, low coverage rate	Kaya-Okur et al., 2019
scChIC-seq	Histone modification	Low throughput, low coverage rate	Ku et al., 2019
scATAC-seq	Chromatin structure	High throughput, high coverage rate	Buenrostro et al., 2015

Furthermore, researchers can now go beyond single-omics studies and can integrate multiple omics in a single cell. For example, the combination of DNA and RNA sequencing by DR-seq (DNA-mRNA sequencing) (Dey et al., 2015) or G&T-seq (genome and transcriptome sequencing) (Macaulay et al., 2015) from the same cell can reveal genomic variations between individual cells, thus explaining changes at the transcription level. Techniques that analyze the epigenome and transcriptome of the same cell have been used to reveal the regulatory role of methylation and chromatin accessibility in gene expression (Angermueller et al., 2016; Hu et al., 2016; Clark et al., 2018), and Hou et al. (2016) have developed scTrio-seq (single-cell triple omics sequencing) that simultaneously obtains genome, DNA methylome, and transcriptome information from a single cell.

There has been an explosion of studies utilizing SCS in recent years due to its remarkable ability compared with traditional sequencing technology to detect new genes that might be missed by bulk sample sequencing and to discern previously unknown cell types (Proserpio and Lonnberg, 2016). In addition, SCS visualization of cell fate transition and possible cell origin has allowed the reconstruction of cell lineage trajectories of many important life activities in cells (Pijuan-Sala et al., 2019), and SCS has proven to be of great importance for illustrating new mechanism of tumorigenesis and metastasis and thus improving the diagnosis and treatment of cancer (Navin et al., 2011). In embryonic organ development, SCS provides genetic information at different stages, thus helping to identify master genes and regulatory signaling pathways in early embryonic development (Tang et al., 2010; Biase et al., 2014) and during the development of the cerebral cortex (Pollen et al., 2014), kidney (Wang P. et al., 2018), lung epithelium (Treutlein et al., 2014), etc. SCS has also promoted the identification of new species of microorganisms and clarified the molecular evolutionary mechanisms and kinetics of infection by obtaining genetic information at the single-cell level (McLean et al., 2013; Combe et al., 2015; Guo et al., 2017b). In studies of the immune system, where the cell subtypes are highly heterogeneous and are even more diversified after gene recombination, SCS has revealed single cell gene-expression networks in immune responses, therefore deepening our understanding of complex immune mechanisms and laying the foundation for advances in immunotherapy (Bossel Ben-Moshe et al., 2019; Rao et al., 2020). In reproduction and heredity studies, SCS is able to detect the dynamic genetic profiles of single germ cells (Hou et al., 2013; Chen et al., 2018; Wang M. et al., 2018) and embryonic cells (Wagner et al., 2018), which is significant for understanding germ cell genesis, alternative splicing patterns, and the key regulators of these processes. SCS also shows promise in reproductive disorders by improving prenatal and preimplantation genetic diagnosis (Li et al., 2017). SCS has also stimulated stem cell research, and embryonic stem cells and induced pluripotent stem cells have been traced by SCS to identify the regulators of embryonic and organ primordium outgrowth (Ealy et al., 2016). Various tissue-specific stem cells have also been explored with SCS, including hematopoietic stem cells (Kowalczyk et al., 2015), intestinal epithelium stem cells (Grun et al., 2015), lung epithelium stem cells (Ikonomou et al., 2020), neural stem cells (Llorens-Bobadilla et al., 2015), and germline stem cells (Guo F. et al., 2015). The single cell genetic patterns provide us with a more comprehensive means of evaluating the dynamic regulatory networks of progenitor cells *in vivo*, and this has helped to clarify the developmental process and has led to modifications of *in vitro* induction protocols. Broad applications of SCS in diverse fields of basic research are summarized in Figure 1C.

## Single-Cell Sequencing in Inner Ear Cell Heterogeneity

**FIGURE 2:**
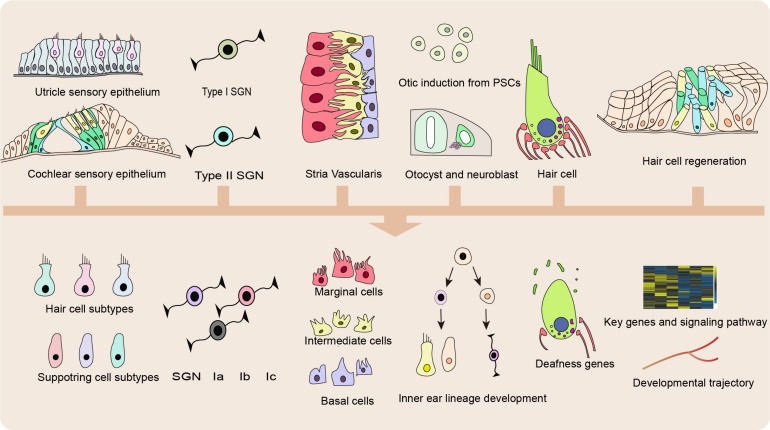
Applications of SCS in basic research. Applications of single cell sequencing (SCS) in the inner ear enable recognition of previously unknown cell subtype and novel marker genes of hair cells (HCs), supporting cells (SCs), spiral ganglion neurons (SGNs), and cells in the stria vascularis (SV), identification of unappreciated exons and splicing diversity of genetic deafness and reconstruction of developmental trajectory and regeneration process.

Hair cells in the inner ear function in transducing the sound waves into electric signals (Hilding, 1953; Zwislocki, 1975; Balak et al., 1990); while SCs function in supporting the HCs and providing the potential pool for HC regeneration (Corwin and Cotanche, 1988; Balak et al., 1990). Damage from a variety of sources can impair HC function, including mutations in deafness genes, ototoxic drugs, aging, chronic cochlear infections, and noise overexposure (Wright, 1973; Cotanche et al., 1987; Hashino et al., 1991; Jiang et al., 2018). As shown in Figure 2, SCS enables the analysis of vast amounts of genetic data simultaneously at single-cell resolution with unbiased cell clustering, thus leading to the identification of novel markers for inner ear resident cells and new cell subtypes of type II extrastriolar HCs, cochlear supporting cells (SCs), neuromast SCs, and spiral ganglion neurons (SGNs) (Burns et al., 2015; Shrestha et al., 2018).

### Heterogeneity of the Sensory Epithelium

Based on single cell transcriptional profiles of postnatal day (P)1 mouse utricular epithelium, Burns et al. (2015) used known markers to identify three major groups [*Gata2*, *Lmx1a*, *Slc26a4*, and *Cldn8* in transitional epithelial cells (TECs); *Otog*, *Otoa*, *Jag1*, *Hes1*, and *Slc1a3* in SCs; *Myo7a*, *Pou4f3*, *Otof*, *Calb2*, and *Ptprq* in HCs] and subdivided them into seven subclusters following principal component analysis-based reduction. TECs contained one cluster, whereas SCs comprised two clusters and HCs consisted of four clusters. In unbiased trajectory analysis, HC.i appeared to be in a transitional state between SC.ii and HC.iii–iv, which is consistent with a study confirming the development of HCs from SCs (Wang et al., 2015). Surprisingly, HC.ii was assigned between TECs and HC.iii–iv, which suggested novel TEC-like progenitor cells located outside the sensory epithelium. In addition, striolar HCs and SCs seem to be distinct cell subtypes compared with their counterparts in the extrastriolar region (Wang et al., 2015; Li et al., 2016). Striolar HCs express high levels of oncomodulin and clusterin, while striolar SCs distinctly express the otolithic membrane glycoprotein beta-tectorin, the transcription factor Gata3, and the retinoic acid-inactivating protein Cyp26b1. In addition, P1 striolar SCs have a greater propensity to regenerate into HCs in response to Notch inhibition. Together, these results indicate that the striola is a molecularly distinct region within the utricle. Another study analyzed single utricular HC data from P1, P12, and P100 mice and identified *Spp1* as a specific marker for Type I HCs and *Mapt* and *Anxa4* as markers for Type II HCs (McInturff et al., 2018). In addition, Ellwanger et al. (2018) identified two classes of type II extrastriolar HCs based on the discrepant expression of *ATP2B2*, *CCDC50*, *MYO1H*, *TMC2*, and *TNNC2* (Ellwanger et al., 2018).

Burns et al. (2015) adopted a similar process to analyze the single cell transcriptomes of P1 cochlear epithelium tissue and identified four major clusters of cells – HCs, SCs, medial non-sensory cells (NSC.i) and lateral non-sensory cells (NSC.ii). *Rasd2*, *Anxa4*, and *Pcp4* were found to be novel markers for HCs, and *Cdh4* and *Mia1* were identified as medial SC markers and *Cntn1* as a lateral SC marker. Although genetic heterogeneity within the clusters indicated possible cell subtypes, definitive conclusion was not available in Burns’s study because of the limited sample size, and this highlights the importance of the power of sequencing (Burns et al., 2015; McInturff et al., 2018). Hoa et al. (2020) collected single cell transcriptomes of cochlear SCs and categorized adult cochlear SCs into two subclusters that differentially expressed *S100a6*, *Pla2g7*, *Tuba1b*, and *Spry2. S100a6* and *Pla2g7* were identified as medial SC markers, and *Tuba1b* and *Spry2* were identified as lateral SC markers.

Comparisons of gene expression between inner HCs (IHCs) and outer HCs (OHCs) provide a deeper understanding of these cells’ unique functions. Using AUC rankings, *Ocm* and *Sri* were identified as the top two most upregulated genes in OHCs, both of which are associated with calcium regulation (Ranum et al., 2019). Expressed predominantly by OHCs, *Ocm* encodes Oncomodulin, a calcium-buffering protein that is essential for mechanoelectric transduction and electromotility amplification (Simmons et al., 2010). *Sri* encodes sorcin, a regulator of calcium-based excitation-contraction (Fowler et al., 2008). OHCs express Ca2+ release channels/ryanodine receptors in a pattern similar to cardiac myocytes, which raises the possibility that Ca2+ plays a similar role in regulating OHC motility (Grant et al., 2006). The expression of other components required for calcium-mediated excitation-contraction in the OHC transcriptome is consistent with this hypothesis and suggests a unique requirement for the tight regulation of calcium in these cells (Ranum et al., 2019).

Compared with mammals, non-mammalian vertebrates constantly regenerate sensory HCs in lateral line organs in order to replace senescent or injured cells, and this occurs through SC proliferation and differentiation (Steiner et al., 2014; Romero-Carvajal et al., 2015; Chen et al., 2017; Zhang et al., 2018, 2019). Regular RNA-Seq has been extensively used in analyzing the proliferation and differentiation ability of SCs (Waqas et al., 2016a; Cheng et al., 2017; Zhang et al., 2017; You et al., 2018), and scRNA-seq was used to identify quiescent and activated stem cell populations and their spatial arrangements in zebrafish neuromasts (Lush et al., 2019). The SC subtypes included central SCs (*Lfng*, *Ebf3a*, *Gata2a/b*, and *Slc1a3a/Glasta*), anterior-posterior (A/P) SCs (*Cx44.2*, *Fap*, *Fgf10a*, and *Hmx2*), dorsal-ventral (D/V) amplifying SCs (S*ost*, *Fsta*, *Srrt/Ars*, *Six2b*, and *Adcyap1b*), and dividing/differentiating SCs (*Dld*, *Her4.1*, *Pcna*, and *Atoh1b*). Dividing/differentiating SCs were considered to be an HC progenitors cluster in the HC lineage with young HCs and mature HCs, which were marked by *Atoh1b* and *Tekt3*, respectively. Mantle cells had enriched expression of *Cldne*, *Crb3b*, *Crip1*, and *Cts12*. The authors then mapped the amplifying divisions to D/V poles and the differentiating divisions to the center and thus identified distinct locations of two SC lineages, while cells in the A/P poles were relatively quiescent. Along this timeline, cells lose their expression of SC and stem cell genes and take on HC-specific markers. Genes related to translation, cell cycle regulation, and Notch signaling (*Dla*, *Dlb*, *Dlc*, *Dld*) were downregulated, suggesting their roles in early developmental stages rather than subsequent differentiation stages.

### Heterogeneity of Cochlear SGNs

After mechanical stimulation of auditory HCs, sound information is conveyed to the dendrites of SGNs via synaptic vesicles containing the neurotransmitter glutamate (Eybalin et al., 1996; Yan et al., 2018; Guo et al., 2019; Liu et al., 2019). The perception of auditory information – including sound frequency, intensity, timbre, and pitch – is a complex process that appears to be associated with SGN diversity (Pfeiffer and Kiang, 1965; Sachs et al., 1974; Liberman, 1982; Temchin, 1988). Based on recent studies of SGN single-cell transcriptomes, three novel subclasses of type I neurons have been identified (Petitpre et al., 2018; Shrestha et al., 2018; Sun et al., 2018). The clustering of type I SGN subtypes in the three studies used almost identical gene sets and obtained similar transcriptional profiles, but the authors named the three subtypes differently. Genes were found to enriched in each subtype, including *CALB2*, *Trim54*, *Rxrg* in type Ia neurons; *CALB1*, *Runx1*, and *Ttn* in type Ib neurons; and *Pou4f1*, *Lypd1*, *Grm8*, *Kcnc2*, *Lypd1*, and *Runx1* in type Ic neurons (Petitpre et al., 2018; Shrestha et al., 2018; Sun et al., 2018). Shrestha et al. (2018) examined genes involved in synaptic transmission and electrophysiological properties. The expression of AMPA-type glutamate receptor subunit *Grik4*, metabotropic receptor subunit *Grm8*, and dopamine receptor subunit *Drd1* were highly increased in Ic SGNs, and the cholinergic receptor subunits *Chrna1* and *Chrna4* were enriched in Ia SGNs. As for the genes encoding K^+^ channel subunits, *Kcnq4* was detected in Ia SGNs, while *Kcnd2* and *Kcnip2* were expressed in Ib SGNs and *Kcnj9* was expressed in Ic SGNs. *Cacna1b*, *Cacna1h*, and *Cacna2d1* are voltage-gated Ca^2+^ channels that were enriched in Ia SGNs. The leaky sodium channel *Nalcn* was detected tin Ib and Ic SGNs, and the voltage-gated sodium channel subunit *Scn2b* was expressed in an increasing gradient from Ia to Ic SGNs. Based on a previous study that SGNs projecting to different positions along the basolateral surface of IHCs differ in spontaneous firing rate (SR) (Liberman, 1982), Shrestha assumed that type Ia SGNs that projected onto the pillar side of the IHC and formed synapses with small ribbons corresponded to high-SR fibers, that Ib SGNs on the middle of the basolateral surface with medium ribbons corresponded to medium-SR fibers, and that Ic SGNs that projected onto the modiolar side with small ribbons corresponded to low-SR fibers (Shrestha et al., 2018). In addition, enriched gene sets for mitochondrial function and neurofilament formation in type Ia SGNs were consistent with increased mitochondrial content and fiber thickness in high-SR neurons (Sun et al., 2018). They also found tonotopic differences in functionally relevant genes within these subpopulations. *Efna1*, for example, was condensed in the middle and base compared with the apex within Ib SGNs and was enriched in Ia and Ic SGNs at the base (Shrestha et al., 2018). The transcriptional profiles and anatomical arrangements, combined with their electrophysiological properties, suggested that the heterogeneous neural populations were involved in encoding of sound intensities and for maintaining of hearing discrimination in noisy environments (Petitpre et al., 2018). This diversity of cochlear neurons is established at birth, followed by refinement over the first postnatal week that requires intact HC mechanotransduction activity (Shrestha et al., 2018; Sun et al., 2018).

### Heterogeneity of the Stria Vascularis

Hair cell mechanotransduction requires high-K^+^ endolymph and positive endocochlear potential (EP), both of which are generated by the stria vascularis (SV), a non-sensory epithelial tissue located on the lateral side of the cochlear duct (Wangemann, 2002; Patuzzi, 2011). Marginal cells (MCs), intermediate cells (ICs), and basal cells (BCs) have been identified in the SV and are indispensable for endolymph ionic homeostasis and EP maintenance.

Korrapati et al. (2019) performed single cell RNA-seq and single nucleus RNA-seq to investigate SV cell populations. Known markers, including *Kcne1* and *Kcnq1* for MCs, *Cd44* and *Met* for ICs, *Cldn11* and *Tjp1* for BCs, and *Slc26a4* for spindle/root cells, were used to define cell identities after unbiased clustering. Gene ontology (GO) analysis showed that MCs had enriched expression of genes involved in positive regulation of K^+^ transport, Ca^2+^-transporting ATPase activity, and G-protein–coupled receptor complex formation, that ICs exhibited high expression of H^+^-transporting ATPase activity, neutrophil degranulation, and interleukin-28 receptor complex formation, and that BCs expressed genes involved in rhodopsin expression regulation and platelet alpha granule lumen. The transcriptional profiles of the SV cell populations suggested roles for MCs and ICs in ion homeostasis maintenance and possible interactions between SV cells (ICs and BCs) and the immune system. In addition, novel markers, including *Abcg1* and *Heyl* in MCs, *Nrp2* and *Kcnj13* in ICs, *Sox8* and *Nr2f2* in BCs, and *P2rx2* and *Kcnj16* in spindle/root cells were discovered and confirmed by smFISH (single molecule fluorescence *in situ* hybridization).

## Single-Cell Sequencing in Inner Ear Development

Generation of the entire otic lineage from non-neural ectoderm (NNE) through pre- placodal ectoderm (PPE) and the posterior placode fates remains elusive, but *in vitro* studies have contributed to understanding the molecular mechanism of early otic lineage formation. Ealy et al. (2016) used human embryonic stem cells and induced pluripotent stem cells to test conditions for stepwise induction of NNE to the otic lineage, and the process was monitored by SCS. They successfully developed a protocol that combined BMP, Wnt, and FGF signaling regulations with retinoic acid treatment to generate a lineage that chronologically expressed markers of NNE (*Dlx3*, *Dlx5*, *Dlx6*, *Tfap2a*, *Tfap2c*, *Gata3*, and *Gata2*), PPE (*Eya1*, *Eya2*, *Six1*, and *Six4*), and ultimately of the otic lineage (*Myosin7a*, *Foxi3*, *Fbxo2*, *Pax2*, and *Pax8*). This breakthrough showed the potential of applying SCS in the refinement of the cell guidance protocol.

The otocyst is regarded as the origin for almost all cells of the inner ear, including sensory epithelial cells and neurons. Durruthy-Durruthy et al. (2014) conducted scRNA-seq of cells from mouse otocysts and neuroblasts at embryonic day (E)10.5, which is the developmental stage at which delaminating and migrating neuroblast cells coexist. Clusters identified by known markers included early neuroblasts (by *Neurog1* and *Fgf3*), post-delaminated neuroblasts (by *Isl1*, *Neurod1*, and *Eya1*), ventral otocysts (by *Lfng*, *Sox2*, *Pax2*, and *Gli1*), and dorsal otocysts (by *Bmp4*, *Dlx5*, *Gata2*, and *Oc90*). Because neuroblasts are committed to their lineage, repressed Shh and Wnt signaling was observed, as shown by downregulation of Shh pathway genes (*Smo* and *Ptc2* and *Gli3*) and Wnt pathway genes (*Fzd2*, *Fzd7*, *Fzd8*, and *Axin2*). Fgf signaling was also downregulated and Fgf gene (*Fgf8* and *Fgf10*) and *Fgfr1* expression was decreased and *Fgfr1* antagonist *Spry2* expression was increased along the axis of development. However, Notch signaling showed a bimodal distribution, and expression of *Dll1*, *Jag1*, *Notch2*, *Hes1*, *Hes5*, and *Hey2* declined while expression of *Jag2* and *Hey1* increased toward the later stages. In addition, two distinct cell populations were identified within the late neuroblasts that expressed *Foxg1* and *Jag2* asymmetrically, which implied the separation of cochlear and vestibular ganglion neurons (Lu et al., 2011; He et al., 2019, 2020). In the otocysts, Notch signaling was mapped to the dorso-anterior side (*Notch2*) and ventro-anterior side (*Hes1*, *Hey1*, and *Hey2*), and the Shh receptor gene *Ptc2* and effector gene *Gli3* were expressed in cells located in the ventral part of otocysts. A small group of cells in the ventral otocyst cell cluster were identified as potential prosensory predecessors due to their enriched expression of prosensory markers (*Sox2*, *Jag1*, and *Lfng*) and weak expression of the early neuroblast marker *Neurog1*. Signaling pathway analysis of this subcluster revealed active Notch (*Hey1*, *Hey2*, *Hes1*, *Hes5*, *Dll1*, *Jag1*, and *Notch2*) and Shh signaling (*Gli2*, *Gli3*, and *Smo*) activity and autonomously regulated Fgf signaling (*Fgf3*, *Fgf10*, *Spry1*, and *Spry2*), indicating the complexity of the signals that need to be integrated to induce the prosensory lineage. *Fbxo2* and *Otol1* were otic-specific candidate genes in the heterogeneous area that gives rise to the prosensory domains.

The endolymphatic sac forms at E10 and is the earliest structure to arise from the otocyst, and it is important in fluid resorption during inner ear development (Morsli et al., 1998; Raft et al., 2014). The major cell types in the endolymphatic sac are ribosome-rich cells (RRCs) and mitochondria-rich cells (MRCs) (Dahlmann and von During, 1995). Honda et al. (2017) conducted single-cell RNA-seq of the mouse endolymphatic sac from E12.5 to P30. Early RRCs predominantly expressed genes involved in proteins secretion (*Dmkn*, *Clu*, *Igsf1*, and *Cyr61*), while mature RRCs expressed genes related to innate immunity (*Lcn2*, *Slpi*, and *Serping1*). In contrast, MRCs expressed high levels of genes related to ion transport (*Slc26a4*, *Atp6v1b1*, *Clcnkb*, *Slc4a9*, *Kcnma1*, and *Slc34a2*). These results confirmed that embryonic endolymphatic sac fluid resorption is carried out by MRCs and is dependent on the anion exchanger SLC26A4.

After cell fate commitment, primitive cells undergo a series of changes in gene expression and cellular morphology to become mature cells. McInturff et al. (2018) identified new markers for utricular HCs (*Spp1* for Type I HCs and *Mapt* and *Anxa4* for Type II HCs), and they traced these genes to study the temporal and spatial development of utricular HCs. They found that type I HCs develop from the posterior-medial side to the anterior-lateral side and that type II HCs initially develop in the striolar region and extend to the periphery. Marked by *Spp1*, 90% of type I HCs arise between E11.5 and E14.5, while almost all type II HCs develop postnatally.

In HCs, hair bundle assembly and protrusion are prerequisites for their mechanotransduction function (Ó Maoiléidigh and Ricci, 2019). Although four stages of developing bundles have been defined according to their structural pattern as pre-growth, initial growth, widening, and secondary growth (Tilney et al., 1992), the molecular basis for this division has remained poorly understood. Using SCS, Zhu et al. (2019) found that utricle hair bundle assembly and growth requires regulation of local Ca^2+^ concentrations. The Ca^2+^ regulator *Calb2* and the calcium pump *Atp2b2* were shown to be important for stereocilia growth because the highest level of *Calb2* protein occurred at the onset of the secondary growth and *Aatatp2b2* protein expression peaked during stereocilia widening (Ellwanger et al., 2018). It has been confirmed that HCs use existing monomeric actin to build hair bundles without significant upregulation of actin gene transcription (Tilney and Tilney, 1988). Zhu et al. (2019) reported the use of single-cell proteomics analysis combined with single-cell RNA-seq to study the utricles of E15 chickens. The authors found previously unannotated proteins that were abundant in different populations, such as GSTO1, GPX2, CRABP1, and AK1 in HCs and TMSB4X and AGR3 in SCs. Single cell proteomics revealed that the actin monomer binding protein thymosin b4 (TMSB4X) was abundant in SCs and that its expression decreased as progenitor cells developed into HCs. Comparative single-cell RNA-seq analysis showed that *Tmsb4x* transcripts were downregulated when transcription of *Atoh1* was activated, which suggested that existing monomeric actin is made available for hair bundle assembly through the degradation of *Tmsb4x*.

## Single-Cell Sequencing in Deafness

Numerous variants in genes related to deafness have been identified, thus illustrating the heterogeneous genetic background of deafness (Shearer et al., 2014). Ranum et al. (2019) used single cell full-length reverse transcriptional analysis with long-read sequencing to identify novel exons and to reveal the unappreciated splicing diversity among deafness-associated genes. They analyzed 12 deafness-associated genes and detected 20 unannotated exons, including exon 1B of *Cabp2* and exons 1B, 22B, 9B, and 31B of *Cacna1d*. Their work showed that the heterogeneity and complexity of genetic deafness is greater than previously believed and suggested that current genetic tests for deafness are incomplete.

Single-cell sequencing technology can also be used to demonstrate the mechanism by which genetic changes lead to specific phenotypes. For example, chromodomain helicase DNA binding protein 7 (*Chd7*) has been shown to be involved in early inner ear development (E8.5–E10.5) by regulating ATP-dependent nucleosome remodeling (Adams et al., 2007; Bouazoune and Kingston, 2012), and single cell transcriptomes of E10.5 mouse otocysts showed that loss of *Chd7* leads to the misexpression of neurogenic genes (*Neurog1* and *Neurod1*), the ventral-associated gene *Lfng*, and Notch genes (*Hey1* and *Hey2*) in dorsal otic cells (Durruthy-Durruthy et al., 2018). This indicated that *Chd7* mutant dorsal otic cells aberrantly adopt mixed dorsal and neurogenic fates, probably because of additional Notch signals. Type II transmembrane serine protease (*Tmprss3*) is another gene that is required for normal hearing, and mutations in *Tmprss3* cause rapid cochlear HC loss between P12 and P14 (Fasquelle et al., 2011). Single cell RNA-seq of inner ear organoid cells revealed elevated expression of apoptosis genes and genes associated with the extracellular matrix in *Tmprss3*-knockout hair cells, while *Kcnma1* (which encodes the α subunits of a Ca2+-activated K+ channel), Ca2+-binding protein genes (*Myl1*, *Pvalb*, *Cib2*, *Mylpf*, and *Tnnc2*), and the Ca2+regulator gene *Sln* were downregulated, suggesting the important role for *Tmprss3* in cellular homeostasis (Tang P. C. et al., 2019).

## Single-Cell Sequencing in HC Regeneration

In mammals, the loss of cochlear HCs leads to permanent hearing impairment because these cells only exhibit very limited spontaneous regeneration capacity and only within the first postnatal week, and such regeneration occurs via the mitotic proliferation or transdifferentiation of SCs (Ni et al., 2016; Waqas et al., 2016b; Wu et al., 2016; He et al., 2017; McGovern et al., 2019). Researchers have focused on the genes involved in cell cycle progression in adult cochlear SC transcriptional profiles generated by SCS and found that lateral SCs (pillar and Deiters’ cells) express some of the transcripts that are enriched in Lgr5 + neonatal SCs, including *Cdkn1b*, *Cks1b*, *Mcm3*, *Mdm2*, and *Shc3*, while genes involved in G1/S transition are more preferentially expressed in adult cochlear SCs (Hoa et al., 2020). GO molecular function and cellular component analysis indicated that the inability of adult cochlear SCs to reenter the cell cycle was related to cyclin-dependent protein serine/threonine kinase activity and condensed chromatin at the centromere.

Overexpression of *Atoh1* can induce SCs to differentiate into ectopic HCs (Zheng and Gao, 2000; Gubbels et al., 2008; Lewis et al., 2012). In work by Yamashita et al. (2018), the *Atoh1*-overexpression transgene was driven by *Fgfr3* (in Deiters’ cells and Pillar cells) in mouse cochleae after tamoxifen-mediated induction at P12, and single-cell transcriptomes were obtained at P12, P26, and P33. *Atoh1*-induced HCs were divided into three groups based on their developmental stages, from initial HCs cells to mature HCs. Along the regeneration trajectory, the expression of HC markers (*Myo6*, *Rasd2*, *Chrna9*, *Pvalb*, *Pou4f3*, and *Chrna10*) increased. *Atoh1* and *Pou4f3* were considered to be key reprogramming factors, while other transcription factors, including *Barhl1* and *Lhx3*, improved the conversion efficiency. In addition, the authors found that *Isl1* boosted the efficiency *Atoh1*-mediated SC to HC conversion.

The Notch and Wnt pathways and their interactions were investigated in HC regeneration, and it was shown that Notch signaling downregulation after HC ablation led to the disinhibition of Wnt signaling and the induction of HC regeneration (Romero-Carvajal et al., 2015). The Fgf pathway is downregulated after HC loss in neuromasts (Jiang et al., 2014). To get deeper insights into the mechanism of HC regeneration driven by these three important signaling pathways, scRNA-seq showed that *fgf3* in zebrafish is only expressed in central SCs under normal conditions and is downregulated when the overlying HCs die (Lush et al., 2019). Moreover, *fgf3-null* mutant zebrafish neuromast cells showed that the Wnt inhibitor *sost* was downregulated and the Wnt target gene *wnt10a* was upregulated, indicating that Wnt signaling is activated in *fgf3*-null mutants. The authors demonstrated that loss of *fgf3* in central SCs led to increased HC regeneration independent of Notch signaling likely through fgfr1a and fgfr2 receptors.

## Conclusion and Future Direction

Over the past decade, SCS technologies have emerged as powerful tools for dissecting cellular heterogeneity and for reconstructing developmental lineages in various tissues, and the application of SCS has brought gene expression studies in inner ear cells to an unprecedented level. SCS provides unbiased research methods for analyzing single cell gene-expression characteristics and global cell heterogeneity in the sensory epithelium, the non-sensory epithelium, and the SGNs, thus allowing the identification of novel marker genes and unknown cell subtypes. AUC ranking and GO analysis point to diverse physiological properties among different cell populations and illustrate the different biological functions of each cell type. The use of SCS in the developing inner ear has revealed dynamic transcriptional profiles, varying signaling pathways, and constantly changing cellular states along the developmental axis, and this has led to the reconstruction of cell lineage progression and the discovery of key regulators of these processes. In addition, SCS supports research into the pathogenic mechanisms of deafness and HC regeneration. With the help of full-length sequencing, the identification of unannotated exons and isoforms of deafness-related genes has expanded our knowledge of the genetic basis of deafness and provides a foundation for new advances in gene therapy.

Despite the wide application of SCS methods in inner ear research, SCS technologies still require further development to reduce background noise, amplification errors, and costs and to improve coverage and sequencing depth. Single-cell isolation methods also need improvement in order to avoid genetic changes during dissociation. Thus, there is great room for improvement and development in SCS technologies. We anticipate that the future application of single-cell profiling in the inner ear will shed substantial light on the cellular heterogeneity involved in inner ear development and hearing and balance functions and will improve the accuracy of regenerative therapies. The potential of SCS technology remains to be fully explored, and single-cell sequencing technologies are likely to yield new discoveries in inner ear research in the future.

## Author Contributions

HL and WL designed this systematic review article and present a synthesis of previous researches on Single-cell sequencing in the inner ear. MW and MX went through all the related manuscript. MW, MX, WL, and HL wrote the manuscript. All the authors contributed to the article and approved the submitted version.

## Conflict of Interest

The authors declare that the research was conducted in the absence of any commercial or financial relationships that could be construed as a potential conflict of interest.
